# Assessment of Water Depth Variability and Rice Farming Using Remote Sensing

**DOI:** 10.3390/s25154860

**Published:** 2025-08-07

**Authors:** Rubén Simeón, Constanza Rubio, Antonio Uris, Javier Coronado, Alba Agenjos-Moreno, Alberto San Bautista

**Affiliations:** 1 Centro Valenciano de Estudios sobre el Riego (CVER), Universitat Politècnica de València, Camí de Vera s/n, 46022 València, Spain; rusibro@etsiamn.upv.es (R.S.); jacocam@etsiamn.upv.es (J.C.); alagmo@etsiamn.upv.es (A.A.-M.); asanbau@prv.upv.es (A.S.B.); 2 Centro de Tecnologías Físicas, Universitat Politècnica de València, Camí de Vera s/n, 46022 València, Spain; crubiom@fis.upv.es

**Keywords:** remote sensing, Sentinel-2, rice, vegetation indices, NIR band

## Abstract

Remote sensing is a widely used tool for crop monitoring to improve water management. Rice, a crop traditionally grown under flooded conditions, requires farmers to understand the relationship between crop reflectance, water depth and final yield. This study focused on seven commercial rice fields in 2022 and six in 2023, analyzing the correlations between water depth and Sentinel-2 reflectance over two growing seasons in Valencia, Spain. During the tillering stage across both seasons, water depth showed positive correlations with visible bands and negative correlations with NIR and SWIR bands. There were no correlations with the indices NDVI, GNDVI, NDRE and NDWI. The NIR band showed significant correlations across both seasons, with R^2^ values of 0.69 and 0.71, respectively. In addition, the calculation of NIR anomalies for each field proved to be a good indicator of final yield anomalies. In 2022, anomalies above 10% corresponded to yield deviations above 500 kg·ha^−1^, while in 2023, anomalies above 15% were associated with yield deviations above 1000 kg·ha^−1^. The response of final yield to water level was positive up to average values of 9 cm. The use of the NIR band during the rice crop tillering stage can support farmers in improving irrigation management.

## 1. Introduction

Rice is a staple food for at least half of the world’s population [1], deeply ingrained in the cultures, economies and food security of its production regions. However, it faces significant global challenges due to its high water requirements, impacting both the environment and economy. Fluctuations in water availability directly affect cultivated areas and yields. Rice production in Europe’s central producing countries—Italy, Spain, Greece and Portugal—decreased by 5.96%, 51.23% and 9.97%, respectively, while Portugal experienced an 8.76% increase, in 2024. Drought conditions in Europe have affected rice cultivation, with Spain being the most affected European country. In 2024, Spain’s cultivated area reduced by 42.62% (sowing only 54,500 ha), and the average yield decreased by 13.97%, the lowest value since 2015 [2].

New tools used in monitoring water management and yield modelling in rice crop are focused on remote sensing. Current studies in rice are using remote sensing data to monitor the effects of drought on the crop and to provide information on the impact of stress on yield loss [3], or the influence of flooding [4]. Remote sensing data have been used in rice cultivation to empirically develop new Kc coefficients based on the evolution of crop reflectance throughout the growing session to estimate crop ETc [5]. Studies conducted by de Lima [6] and Bwire [7] related vegetation indices to study the variability in irrigation application and rice growth kinetics and allowed the establishment of guidelines for the crop management of irrigation water. Although the most widely used indices are the Normalized Difference Vegetation Index (NDVI) or the Green Normalized Difference Vegetation Index (GNDVI), the Normalized Difference Red Edge (NDRE) has proven to be an important index supporting agricultural management practices.

Continuous flooding in rice production is a widely used irrigation system in Europe, Japan, South Korea and the United States due to the high water requirements of crop [8]. Water plays a crucial role in rice cultivation in several ways: (a) it acts as a thermal regulator, providing heat to the plant and protecting it from sudden temperature changes; (b) it aids in the transport of dissolved or suspended nutrients; (c) it facilitates oxygen transfer, which is essential for plant growth in flooded conditions and also helps control weed germination [9,10]. Plant growth is limited by the amount of water provided to a field, and consequently, for a specific crop area, by the height of the water layer. The temperature of the irrigation water, which is determined by the height of the water depth, influenced rice seed germination and plant growth rate. Furthermore, the height of the water depth exerts an influence on the germination and growth of weeds, which in turn affects the growth and final yield of the rice plants. Consequently, the regulated provision and surveillance of irrigation water are imperative for the sustainable cultivation of rice [11].

Rice cultivation in Spain primarily takes place in environmentally protected wetlands. These areas are of high ecological value, so managing irrigation water sustainably is essential to improve water use efficiency. The water management follows the strategies proposed by Tinarelli [12] and Osca [13], which involve flooding the crop and making two- or five-day water cuts for weed control and cover fertilization. The authors suggest a water sheet height of 10–15 cm. However, no information is provided regarding the relationship between water depth and the effect on rice growth and final yield. The lack of information and limited water availability highlights the importance of controlling water depth throughout the phenological stage to ensure efficient and sustainable irrigation management.

Therefore, this work presents the hypothesis that it is possible to monitor and model the productive response of the rice crop at different phenological stages using remote sensing data. This work aims to monitor the influence of the height of the water sheet from satellite images (Sentinel-2) and obtain anomaly maps as a function of the reflectance recorded to establish crop management strategies adapted to make decisions in the rice industry.

## 2. Materials and Methods

### 2.1. Location Description

**Figure 1:**
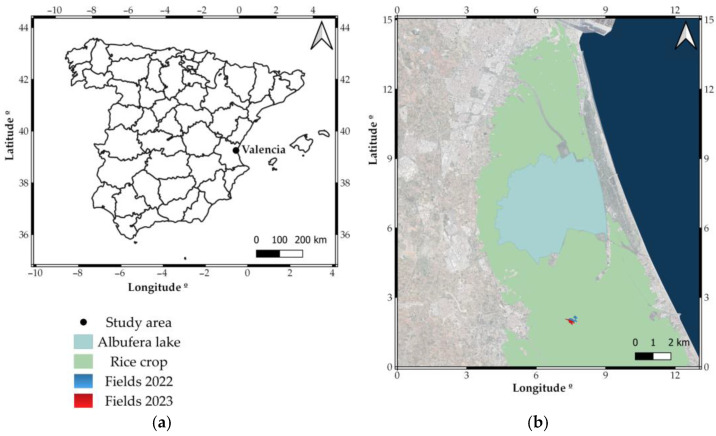
Location of the experiment and the study area. (**a**) Location of Valencia in Comunitat Valenciana, Spain. (**b**) Location of fields around L’Albufera.

The study was carried out during the seasons of 2022 and 2023, in fields located at Sueca, a traditional rice production area in the Albufera Lake of Valencia (Spain), where 8000 ha of rice are located out of the total 15,000 ha of rice cultivation in Valencia. The experimental plots are commercial fields owned by the same farmer—a group of 7 plots in 2022 and 6 plots in 2023, totalling 6.42 and 10.84 hectares (Figure 1).

**Figure 2:**
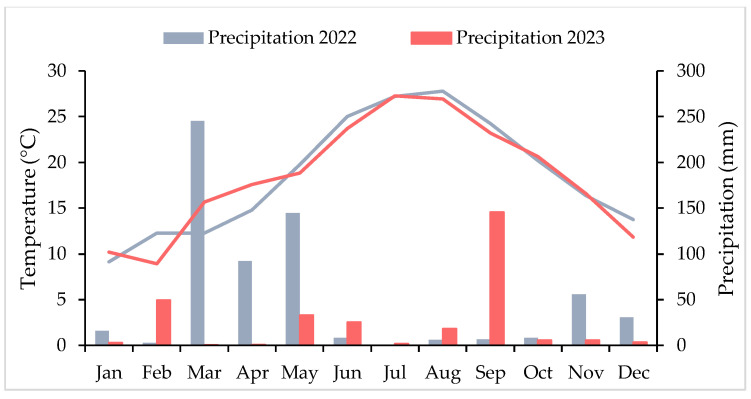
Monthly climogram of the years 2022 and 2023 in the study area. The lines correspond to temperature values, and the bars represent precipitation levels.

The soil is sandy loam with a pH: 7.98 ± 0.01, organic matter content: 3.0 ± 0.02% and electrical conductivity (EC): 3.20 ± 0.01 dS·m^−1^. The total amount of nitrogen was 2938 ± 21 mg·kg^−1^ (air dried basis), available phosphorus: 8.5 ± 0.3 mg·kg^−1^ and available potassium: 0.87 ± 0.01 meq·100 g^−1^. Papadakis’ agroclimatic classification system defines the climate in this area as subtropical Mediterranean with hot and dry summers [14]. Figure 2 shows the mean temperatures and daily precipitation in the 2022 and 2023.

Table 1 shows the values of mean, maximum and minimum temperature (°C), relative humidity (%), radiation (MJ·m^−2^), daily sunshine hours (h) and evapotranspiration (ETo; mm) of the crop calculated according to Allen et al. [15]. The comparison was performed for the entire crop season and for first 100 days after sowing (DAS) each 30-day period.

Sowing was delayed until June in 2022 due to rainfall in April and May (Table 2), while in 2023 it was sown in May. Table 2 shows the variation in Accumulated Growing Degree Days (AGDD); average daily maximum (T max) and minimum temperatures (T min) minus a base temperature (T base) (in the case of rice is equal to 10 °C) and the Vapour Pressure Deficit (VPD) values, every 30 DAS. The climatic conditions shown in Table 1 and Table 2, justify how the year 2022 was warmer and drier in the first DAS and thus conditioned the rapid growth of the crop in the first stages.

### 2.2. Design of the Experiment

In all the plots, the same crop management of all the cultivation tasks has been carried out, including irrigation and fertilization. Although all plots received water from the same canal system, water depth varied naturally due to microtopographic differences and farmer practices. This observational setup allows for studying real-world variability but limits causal interpretation. So, the flooding and drying of the fields are performed on the same date. Table 3 shows the sowing and harvesting dates for the study plots in 2022 and 2023. The rice variety cultivated was ‘JSendra’, a round grain rice, sub-species *Oryza sativa* L. ssp. *Japonica* sowing was conducted under flooded conditions (“water seeding”) at 2–3 cm of water sheet and at a dose of 215 kg·ha^−1^.

Once the crop was planted, the water level in the plots was increased, maintaining the level set by the farmer in each plot. The soil remained flooded for most of the crop cycle, and Table 4 shows the dates of drying carried out.

Nutritional plant, weed and pest management followed common grower practices and recommendations of Osca [13]. Nitrogen fertilizer was applied two times: 140 UF for base fertilizer (applied before flooding) and 30 UF for panicle fertilizer.

Harvesting of all the plots studied was carried out in October of both years. Yield values were obtained by the Deutz-Fahr B9306 TSB combine harvester (Deutz-Fahr, Bavaria, Germany) fitted with Yield Trakk software (Topcon company, Tokyo, Japan), capable of measuring crop yield in real time. The cutting width used by the combine was 7.6 m, providing continuous yield data (approximately every metre travelled). The processing of these yield maps was carried out according to the methodology described in Fita et al. [16], the yield map was transferred to a grid composed of 100 m^2^ polygons (coinciding with the spatial resolution of the satellite), obtaining the average of each of them. All computational transformation and data processing was performed with the QGIS 3.10.14 software [17].

### 2.3. Remote Sensing Data

The images were obtained from the Sentinel-2 constellation (downloaded from the official website of the Copernicus programme). The T30SYJ tile (level 2A) has been used to obtain the images, in a period from May to October of both seasons. Data processing was also carried out using QGIS software.

All the available spectral bands at a 10 m and 20 m spatial resolution and temporal resolution of 5 days were studied, but only for cloud-free days images (Table 5); the main characteristics of each band are shown in Table 6. In addition to the surface reflectance in the spectral bands, these were combined into the vegetation indices (VIs) shown in Table 7.

Table 5 shows the recording dates of the satellite data and their correspondence with the days after planting, together with the main phenological stage of the plants, using the BBCH (Biologische Bundesanstalt, Bundessortenamt y CHemische Industrie) scale as a reference [23].

The satellite data were processed according to the methodology proposed by San Bautista et al. [24], which allows crop monitoring based on reflectance values. In this way, the following data processing has been carried out:

- Spectral evolution of the Red and NIR bands, and average NDVI values for each plot in the years 2022 and 2023.
- Study of correlations between water height and Sentinel-2 bands.
- Study of anomalies in NIR reflectance values and performance data.

### 2.4. Measurement of Water Height

The process of measuring the height of the water sheet was carried out at 35 DDS, on 12 July 2022 and 19 June 2023 (coinciding with the phenological stage: 2—tillering). This date was chosen because it is the moment when the crop has a constant and regular water height over time (it is not influenced by droughts); furthermore, it has been set in accordance with the study carried out by Franch et al. [25], which identifies this period as a critical time for achieving high yields (tillering phase). In addition, insufficient water uptake during this stage can inhibit tillering, leading to lower final yield [26,27]. Consequently, water management is a key decision in rice farming to improve tillering, increase final yield and correct field anomalies. A representative sample of each plot was chosen by measuring the height in 10% of the Sentinel-2 pixels of each plot, selecting the pixels randomly and geo-referencing their location with GPS. A 30 cm ruler was used to measure the points, with 6 repetitions per control point. Interpolation was performed via IDW (Inverse Distance Weighted) in QGIS. While this method provides a practical estimate, potential interpolation error should be considered in pixel-level analyses. The formula used is as follows:

(5) $$Zp=\;\frac{\sum_{i=1}^{n}\left(\frac{z_{i}}{d_{i}^{p}}\right)}{\sum_{i=1}^{n}\left(\frac{1}{d_{i}^{p}}\right)}$$

where

*Zp*: estimated value for unmeasured *p*-point;
*n*: number of points used in interpolation;
*i*: measured value of point *i*;
*z_i_*: value of the coordinate at the *i*-th point;
$d_{i}^{p}$: distance between the *i*-th point and the known point *i*, raised to the power (*p* = 2).

### 2.5. Statistical Analysis

Statistics were run using R software (version 4.2.3). The ANOVA statistic was used to study the differences in water depth and crop yield between the study plots, correlation analysis was used to find out the strength of the relationship (r) and whether it is positive or negative, and the coefficient of determination (R^2^) of the models was proposed for estimating the height of the sheet of water. The separation of means was carried out with the Least Significant Difference (LSD) statistical test with a *p* < 0.05.

## 3. Results

The results of the experiment are presented to evaluate (1) crop monitoring using Sentinel-2 imagery over two growing seasons to assess whether differences in growth kinetics could influence the analysis; (2) the correlation between spectral bands and vegetation indices with field-measured water depth, in order to identify key physical parameters and phenological stages for estimating water depth via remote sensing; (3) the parcel-level anomaly in the near-infrared (NIR) band and its relationship with final yield, to quantify yield variability using Sentinel-2 data; and (4) the direct influence of water depth on crop yield, to determine which water levels are associated with higher productivity.

### 3.1. Spectral Reflectance

**Figure 3:**
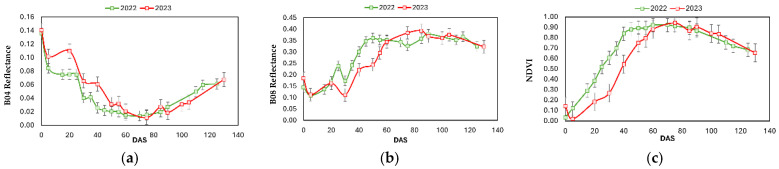
Evolution of red reflectance (B04) (**a**), NIR (B08) (**b**) and NDVI (**c**). Vertical bars indicate standard errors.

To assess interannual crop growth dynamics, the average spectral response of the plots was analyzed over the growing season based on days after sowing (DAS). Reflectance values from B04 (Red) and B08 (NIR) bands and the NDVI were used to monitor vegetation development. This temporal analysis, presented in Figure 3, highlights differences in canopy vigour between seasons. The reflectance values of both bands decrease after flooding (0 DAS), presenting previously higher values (dry soil). In the first 30 days of cultivation, due to the water management (first drying of the crop), a different behaviour in the spectral evolution of bands and NDVI can be observed. Thus, it is from this moment when in some plots, some anomalies could be identified, caused by a different development of the crop.

Crop growth resulted in a decrease in reflectance in the Red band, until the crop flowered (78–85 DAS). During gleaning and at later stages, the rice plant started to lose the green colouring (chlorophyll). This process was accelerated during the maturation and senescence of the crop, coinciding with the increase in the value of red reflectance.

Reflectance in the NIR showed an increasing value since 30 DAS, in vegetative phase, where the highest Aboveground Biomass (AGB) is produced. The values increased until the crop reached the reproductive phase in its last stages (110 DAS). Reflectance values in 2022 were higher during the first 60 DAS, indicating a high biomass growth rate. Since 90 DAS, the loss of NIR value started, with very similar values between years.

NDVI values followed the characteristic evolution of rice cultivation, with a constant increase after planting, with maximum values around 60 DAS in 2022 and 80 DAS in 2023. The displacement of the NDVI evolution in 2023 to the right of the graph indicated the time differences in growth and maturation between the two years.

### 3.2. Study of Correlations Between the Height of the Water Surface and Sentinel-2 Bands

**Figure 4:**
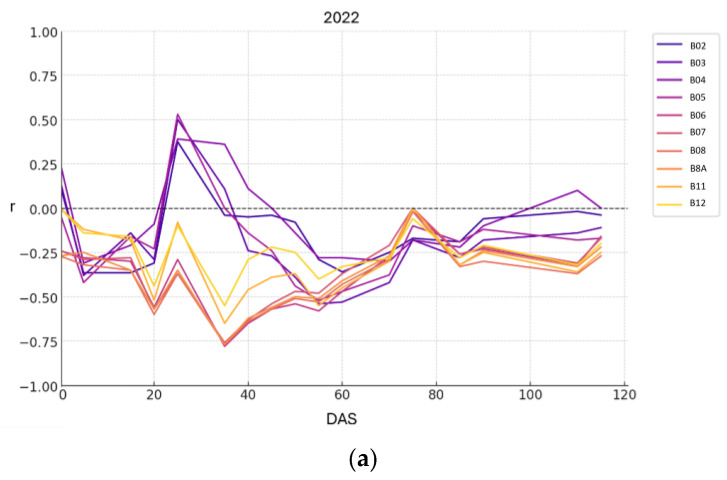

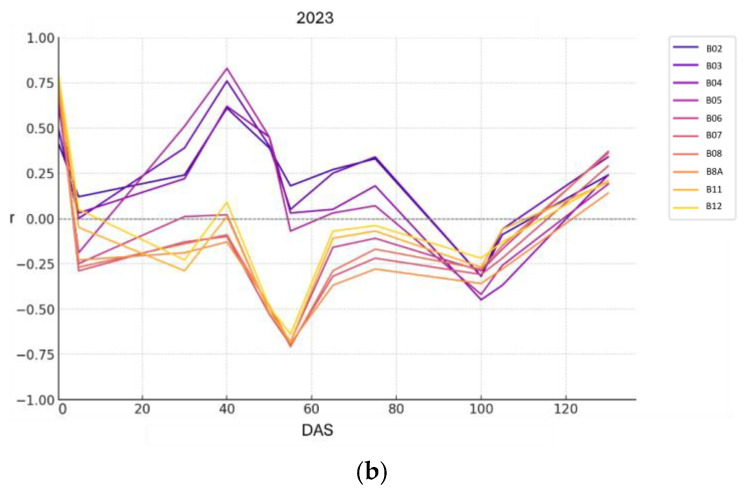
Temporal evolution of the correlation coefficients (r) between measured water depth in the field and Sentinel-2 spectral bands across the 2022 (**a**) and 2023 (**b**) growing seasons, expressed as DAS.

The correlation between water depth and the Sentinel-2 spectral bands was analyzed across all acquisition dates (expressed as DAS) for the 2022 and 2023 growing seasons, as illustrated in Figure 4 (panels (a) and (b), respectively).

It was observed that at 0 DAS, the correlations for 2023 were high; however, the values did not follow a linear trend. In both study years, the correlations followed similar evolutions, represented at DAS. The visible bands (B02, B03, B04) and the B05 band followed a similar and opposite trend to the NIR and SWIR bands (B06, B07, B08, B11 and B12). Until the end of tillering (40 and 55 DAS in 2022 and 2023) the correlations were positive for the visible bands and negative for the NIR and SWIR bands. Since flowering stage (60 DAS), in 2022 correlations were negative and close to 0 until the end of the cycle. This evolution was also observed in 2023, up to 100 DAS where correlations were all positive.

**Figure 5:**
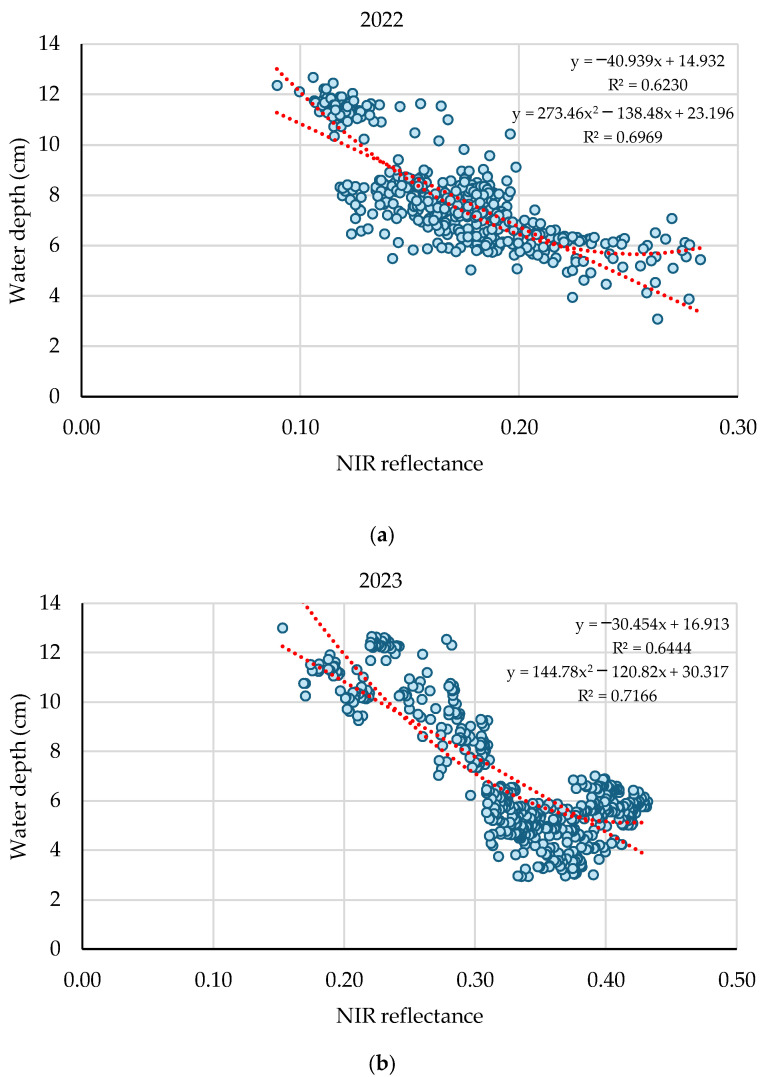
The relationship between NIR reflectance and water depth measured in all pixels of study area in season 2022 (**a**) and 2023 (**b**). The relationship was statistically significant (*p* < 0.05).

The highest correlation values resulted with the NIR band in the tillering season, namely at 35 and 55 DAS, with negative values of r = −0.79 and r = −0.71 in the years 2022 and 2023, respectively (Figure 4). The relationship between NIR reflectance and water depth was analyzed for all pixels within the rice field at 35 and 55 DAS in 2022 and 2023 season (Figure 5a,b). In both years, a clear negative correlation was observed, indicating that higher reflectance values in the NIR region were associated with lower water depths. In 2022, the linear model obtained an R^2^ of 0.623, while the quadratic model slightly improved the fit with an R^2^ of 0.697, suggesting a polynomic behaviour between water depth and NIR reflectance. Similarly, in 2023, the quadratic model also improved over the linear model, with R^2^ values of 0.717 and 0.644, respectively. These results confirm the consistent inverse relationship across both seasons and highlight the potential of NIR reflectance as a reliable indicator of water depth variability in flooded rice fields in the tillering phase. At shallower depths (3–8 cm), small variations in water height produce significant changes in NIR reflectance, due to the greater sensitivity of the spectral response in that region. From approximately 8–10 cm, this response stabilizes, and between 12 and 14 cm it tends to saturate, showing less variability. Therefore, the polynomial trend better captures this transition, especially at the inflexion point of around 8 cm.

Conversely, correlations between vegetation indices and water depth were generally low. The highest R^2^ values were observed for GNDVI at 35 DAS in 2022 (R^2^ = 0.37) and for NDWI at 80 DAS in 2023 (R^2^ = 0.36) (Supplementary Materials, Table S1).

### 3.3. Relationship Between Field-Level NIR Anomalies and Yield Variability

**Figure 6:**
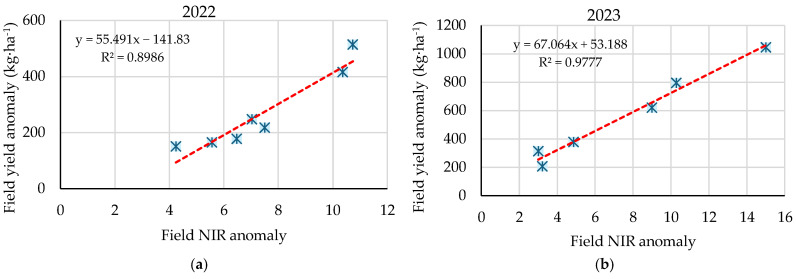
Relationship between NIR reflectance anomaly and final yield anomaly at the field level during the 2022 (**a**) and 2023 (**b**) season.

For each plot, the average of the yield anomalies represented per pixel has been calculated, as well as the average in absolute value of all anomalies in percentage of the NIR at 35 and 55 DAS in 2022 and 2023. The correlation between the two averages is shown in Figure 6. A strong positive linear correlation was observed in both seasons (R^2^ = 0.8986 and R^2^ = 0.9777), indicating that higher deviations in NIR reflectance were strongly associated with higher deviations in grain yield. This suggests that NIR anomalies, derived from satellite imagery, effectively captured intra-seasonal variability in crop yield.

**Figure 7:**
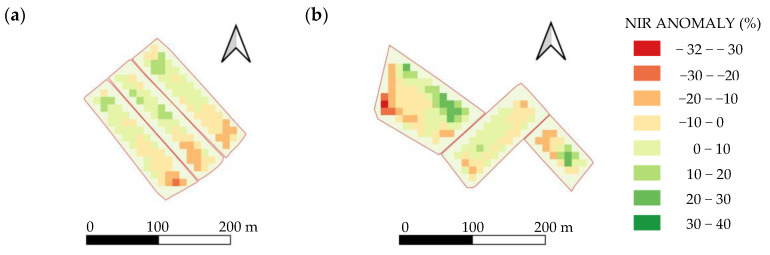
NIR anomaly map at pixel level in fields during the 2022 (**a**) and 2023 (**b**) season.

The linear regression has a slope of 55.49 and 67.06 in the years 2022 and 2023, respectively. Each point of anomaly in the NIR is associated with an average variation of 67.06 kg·ha^−1^ in yield, which represents an increase of 21% with respect to the value obtained in 2022. The main similarity between the two seasons is the strength of the correlation observed, with R^2^ values above 0.89 in both cases. Both graphs show a linear regression model; however, in 2023, the NIR anomalies exhibit higher anomaly, with values reaching up to 15%, compared to a maximum anomaly of 11% recorded in the 2022 season. No anomaly values between 8 and 10% have been recorded in Figure 6a; however, a linear correlation is still present. Figure 7 shows the NIR anomaly per pixel in the tillering phase of the 2022 and 2023 fields.

### 3.4. Influence of Water Depth on Final Yield

#### 3.4.1. Comparison Between Mean Water Height and Yield in the Plots

Table 8 shows the relationship of plots with the mean values of water depth and final yield (kg·ha^−1^) in 2022 and 2023 recorded in the Yield Trakk software of the combine harvester, together with the mean comparison study using the LSD statistical test (*p* < 0.05).

The analysis of variance for the variables water depth and crop yield shows that the plot effect is statistically significant (*p* < 0.01). The statistical differences between the plots allow sufficient variability to be generated to address the proposed modelling.

The study between the two years shows that the average water depth decreased from 8.31 cm in 2022 to 6.94 cm in 2023 and yield increased from 6961.24 kg·ha^−1^ in 2022 to 7325.23 kg·ha^−1^ in 2023. In addition, in 2023, the coefficient of variation in water height and yield also increased compared to the previous year. If the study is analyzed by year, in 2022 there seems to be a positive correlation between water height and yield. Plot 1 recorded the highest depth (11.41 cm) and had the highest yield (7333.5 kg·ha^−1^). Plots with lower depth, such as plot 4 (5.78 cm), showed significantly lower yields (5425.50 kg·ha^−1^). Plots with greater average water depths tended to exhibit higher yields.

In 2023, the relationship between water height and yield did not follow the same trend as in the previous year. Plot 1, with the lowest water depth, had the lowest yield (5988 kg·ha^−1^). However, plots 4 and 5 had the highest mean depth (12.4 cm and 12.22 cm, respectively) but not the highest yields. Plot 2, with a mean depth of 5.98 cm, had the highest yield (9003.42 kg·ha^−1^), with no statistical difference with the yield of plot 6 (8316.22 kg·ha^−1^) which had a mean height of 9.91 cm.

#### 3.4.2. Analysis of the Correlation Between the Water Depth and Yield

**Figure 8:**
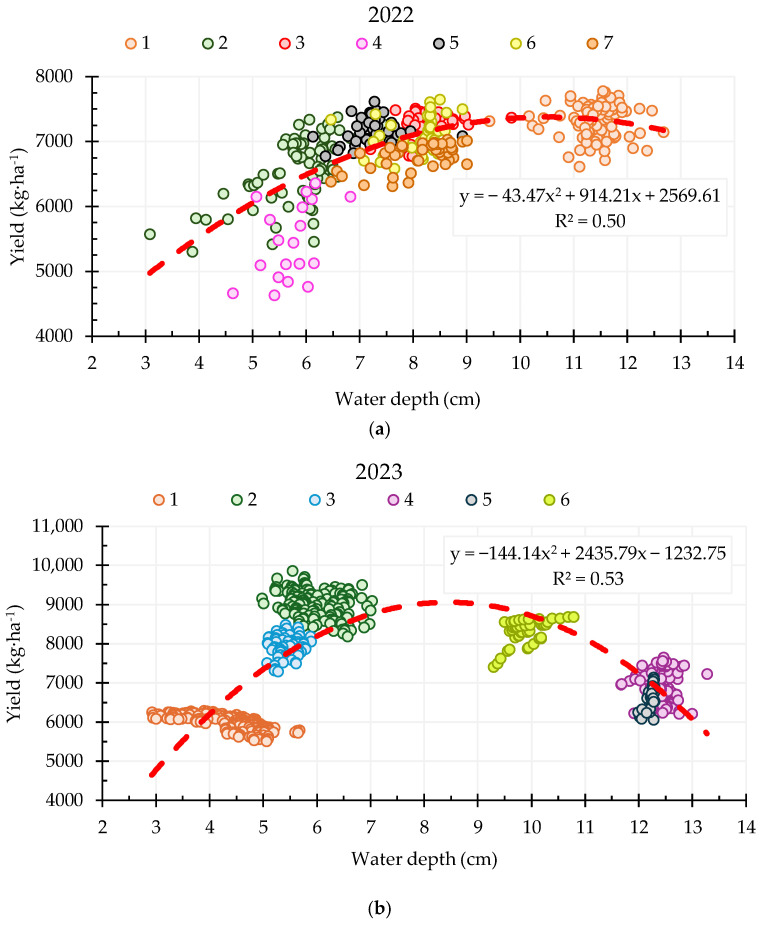
Relationship between water depth and final yield at all pixels of the fields during the 2022 (**a**) and 2023 (**b**) season. The relationship was statistically significant (*p* < 0.05).

Figure 8 shows the evolution of the yield as a function of the height of the water depth, considering the values at pixel level, using a grade 2 polynomial adjustment.

The values obtained for the coefficient of determination were 0.50 and 0.53 considering the yield values of 2022 and 2023, respectively, being *p* < 0.01. This value decreases considering the average value of the yield in both years (R^2^ = 0.19; *p* < 0.01) due to the differences in yields between both years.

For the year 2022 the graph shows a positive polynomial relationship between the height of the water depth and the yield, represented by the adjusted curve. The coefficient of determination is R^2^ = 0.50. It is observed that, in general, as the water depth increases up to 8–11 cm, the yield tends to increase. Plots with water depths in the range of 6 to 9 cm tend to show a consistent increase in yield. This suggests a positive correlation, but with a tendency to stabilize when the water table height reaches values of 11 cm.

The relationship between water depth and yield in the year 2023 shows an inverted parabola shape in the polynomial curve, with a coefficient of determination R^2^ = 0.53. The yield shows an increase with increasing water table height up to an optimum value of 9 cm, and once this optimum height was obtained, the yield started to decrease with increasing water depth.

## 4. Discussion

Based on the remote sensing results, it can be observed that the evolution of reflectance values for the rice crop during the 2022–2023 study seasons followed a characteristic trend for this crop [28,29,30,31,32]. Reflectance in the NIR band is correlated with crop biomass [31]. In both years, the NIR values increased from 30 to 100–110 DAS, corresponding to the vegetative and reproductive phases of the crop, where the greatest accumulation of Aboveground Biomass (AGB) occurs [32]. NDVI is the most frequently employed index in the scientific literature for vegetation assessment [33], as it combines the NIR and Red bands to calculate a value ranging from −1 to 1. This index is highly useful for monitoring the different phenological stages; however, a limitation of NDVI is its tendency to exhibit saturation effects during periods of rapid growth [34]. The rapid evolution and saturation of the NDVI in 2022 allowed us to explain the fast growth rate of the crop.

Across the two years of the study, water depth showed the strongest correlation with NIR values at the tillering stage in both seasons, with R ^2^ = 0.67 and 0.71 following a polynomial adjustment. During the tillering stage of the rice crop, the canopy has not yet fully covered the surface, allowing the soil background—whether dry, moist or covered by a water layer—to significantly influence reflectance values. The water layer in the rice field during the tillering period, if the traditional hyperspectral acquisition time is used, will be interfered with by specular reflection causing spectral pollution [35]. In this context, a decrease in reflectance values across the spectrum with increasing moisture in soils without vegetation is commonly reported in the relevant literature [36,37]. In rice studies, Niel et al. [38] confirm that background water absorption can reduce canopy reflectance compared to individual leaf reflectance when vegetation cover is less than 100%. The reflectance of natural waters, both turbid and clear, can be significantly higher than that of green vegetation in the visible range of the spectrum [39,40]. Green vegetation reflects strongly in the NIR, while the presence of surface water drastically reduces this reflectance due to its high absorption capacity in this spectral region [41,42]. For these reasons, the correlations between water depth and Sentinel-2 bands were positive for visible bands and negative for NIR bands.

In other bands, the study of correlations with the various vegetative indices (NDVI, GNDVI, NDRE and NDWI) did not show correlations with water depth. Scientific bibliography deals with these indices to monitor the flooding of rice plots and to detect production zones. Boschetti et al. [43] described the potential use of visible bands (Green or Red) with the SWIR for detecting flooded areas. Subsequently Albertini et al. [44] evaluated different studies analyzing the potential use of the MNDWI and NDWI for monitoring and detection of water on the land surface. Other studies use EVI and NDFI to establish rice crop cycles in different areas of the world [45]. And although Pedroso de Lima et al. [6] analyzed the variability between plots using drone and satellite vegetative indices and confirmed their potential for irrigation management in the Portuguese growing area, no previous study has analyzed the relationship between crop reflectance, water depth and final productivity. As a result, higher water table levels would improve crop tillering, causing consequent increases in biomass that would translate into higher NIR reflectance values [46] and higher chlorophyll content, which would result in lower reflectance values in the visible bands [47]. In addition to the absolute reflectance values, it would be important to monitor the variability in reflectances in each field, since wide deviations from these values would indicate anomalies and therefore deviations from the crop’s potential yield, given the fact that agricultural management decisions are usually made on a per-field basis [48].

In this context, crop monitoring is essential to ensure optimal growth and development. Therefore, it is important to identify the critical phenological stages to achieve high yields. Abid Nazir et al. [49] identified the end of tillering and flowering as the best phenological stages to develop a linear regression model to predict yield using Sentinel-2 vegetative indices in the production area of Pakistan. Soriano-González et al. [50] reported the highest correlation with final yield at the end of tillering, based on the maximum NDVI and minimum NDWI values. Preliminary studies on Sentinel-2 band reflectance in the same production area (Albufera de Valencia) also identified the tillering stage as showing the highest correlation with final yield [24]. In addition, Fita et al. [16] reported that crop yield had the strongest correlation with NIR reflectance, with higher R^2^ values than those obtained using various vegetation indices. As observed in this study, a 10% anomaly in NIR reflectance during the tillering stage in the 2022 season was associated with a yield reduction of approximately 500 kg·ha^−1^, while a 15% anomaly in 2023 resulted in losses of up to 1046 kg·ha^−1^. In this context, analyzing NIR reflectance variability during the tillering stage could be a valuable tool for preventing deviations in final yield.

In 2022, the highest yields were recorded at water depths of 8 and 11 cm with values of 7244 and 7333 kg·ha^−1^, respectively. These final yield values showed no statistically significant differences, suggesting that yields were maintained from 8 to 13 cm water depth. In 2023, the highest yields were observed at lower depths (6–7 cm), reaching 9003 kg·ha^−1^, followed by yields around 8316 kg·ha^−1^ at 9 cm. However, deeper water levels of 11 to 13.5 cm during the 2023 season resulted in lower yields, averaging around 6700 kg·ha^−1^. These results suggest that the optimum water depth range for maximum productivity in both years was around 9 cm. According to the literature, water depths under continuous flooding systems have shown diverse results. Talpur et al. [51] reported that maintaining 5 cm of water during the vegetative stage and 10 cm during the reproductive and grain filling stages was optimal for maximum yield, while deeper water levels (20 cm) resulted in reduced growth and yield. Similarly, Juraimi et al. [52] recommended a depth of 10 cm combined with longer inundation periods for higher productivity. Chiba et al. [53] conducted field experiments to evaluate different flooding depths (2, 8, 12, 15, 18 cm) and recommended 10 cm as the ideal depth to ensure high grain quality. In the same line, Anbumozhi et al. [54] evaluated flooding depths (0, 3, 6, 9, 12, 15, 18 cm) and found that 9 cm resulted in the highest rice yield. Although most studies converge on 10 cm as the recommended depth, Fan et al. [55], established that 15 cm of flood depth can increase yield production by 4% and 18% compared to 5 cm and 10 cm, and saves 36% of water compared with 20 cm. Therefore, although 9 cm seems to be an optimal value, the ideal depth for rice cultivation depends on the study area, climatic conditions, water quality and availability, field levelling and irrigation system.

Monitoring NIR band using satellite imagery during the tillering stage of rice cultivation could assist farmers to identify areas with higher or lower water depths. Moreover, the generating of NIR anomaly maps at this stage could enable early detection of possible yield deviations, allowing for timely decisions on interventions to minimize productivity losses. This information derived to satellite could be used to dynamically adjust irrigation flow rates, optimize water input/output balance and inform land levelling adjustments for the following season to improve the uniformity of water distribution across fields. In this context, the results of this study provide practical support for farmers to implement more precise water management strategies using easily accessible remote sensing data with field-level applicability.

## 5. Conclusions

The present study explores the applicability of remote sensing techniques for evaluating water depth variability in final rice yield. Based on the remote sensing results, the relationship between reflectance values from Sentinel-2 bands and crop water depth could be analyzed. The visible bands showed positive correlations with water depth, while the NIR reflectance values showed negative correlations. The NIR band (B08) showed the highest correlation values between water depth and reflectance during the tillering stage in both study years. Furthermore, the analysis of anomalies in the NIR values of each pixel relative to the plot average allowed for the early detection of yield deviations in a quantitative manner. These results support the implementation of more precise water management strategies by farmers, using accessible satellite remote sensing data with direct field applicability. Such observations provide accurate characterization of the magnitude of productivity losses associated with spectral deviations within a plot, which is essential for the development of early warning systems and intervention strategies based on remote sensing. Despite the limitations of the study, there are local factors such as field levelling, irrigation systems and crop variety, which may also influence the water distribution and its spectral response and may affect the extrapolation of the models beyond the study area. Nevertheless, the methodology is considered reproducible in other contexts. Future work should focus on validating this approach across different rice-growing regions and integrating higher-resolution imagery or using image fusion techniques to improve spatial resolution, incorporate more remote sensing data to work with more time and incorporate machine learning models for operational early-warning systems.

## Figures and Tables

**Table 1:** Mean (T mean), maximum (T max) and minimum (T min) temperatures, relative humidity, radiation, sunshine hours and ETo every 30 days for all rice seasons in the experimental plot area in 2022 and 2023.

Period of DAS	0–30			30–60			60–90			90–120			120–140		
Year	2022	2023	Desv (%)	2022	2023	Desv (%)	2022	2023	Desv (%)	2022	2023	Desv (%)	2022	2023	Desv (%)
T mean (°C)	22.74	20.06	−11.79	25.94	25.68	−1.02	28.49	27.73	−2.66	26.96	25.83	−4.21	22.61	22.65	0.17
T max (°C)	29.44	25.56	−13.18	32.15	30.88	−3.95	34.38	33.13	−3.65	33.67	31.19	−7.37	28.59	28.71	0.41
T min (°C)	15.67	14.96	−4.55	19.52	20.05	2.72	22.36	22.71	1.53	20.49	20.49	0.01	17.02	17.06	0.22
RH mean (%)	68.17	73.03	7.13	65.62	73.11	11.42	67.48	71.73	6.31	66.47	72.94	9.73	66.61	72.93	9.49
Radiation (MJ·m^−2^)	27.67	22.14	−19.97	26.15	25.31	−3.22	25.31	24.82	−1.96	21.42	20.47	−4.42	15.26	17.20	12.69
Sunshine hours (h)	12.51	11.21	−10.34	12.52	12.01	−4.08	12.16	12.01	−1.19	11.03	10.84	−1.71	9.65	9.97	3.35
ETo (mm)	167.68	130.59	−22.12	168.54	157.84	−6.35	170.46	164.68	−3.39	147.22	133.65	−9.22	66.36	69.13	4.17

**Table 2:** The Accumulated Growing Degree Days (AGDD) and Vapour Pressure Deficit (VPD) each 30 days in rice seasons in experimental plots in 2022 and 2023.

DAS	AGDD			VPD (kPa)		
	2022	2023	Desv %	2022	2023	Desv %
0–30	476	462	−2.96	1.53	1.24	−18.89
30–60	556	529	−4.90	1.69	1.13	−32.89
60–90	539	596	10.54	1.83	1.29	−29.76
90–120	410	541	32.05	1.26	1.11	−12.24
120–140	234	317	35.48	0.82	0.99	20.80

**Table 3:** Dates of sow and harvest for 2022 and 2023 season.

	2022	2023
Sowing	9 June	15 May
Harvest	22 October	6 October

**Table 4:** Dates about drying seasons in 2022 and 2023.

Year	First Drying	Second Drying	Final Drying
2022	21 June	19 June	20 September
2023	30 May	15 June	10 September

**Table 5:** Dates studied from Sentinel-2 for DAS and phenological state of BBCH observed in the fields in 2022 and 2023.

Date 2022	DAS	BBCH Scale	Date 2023	DAS	BBCH Scale
9 June	0	0—Germination	15 May	0	0—Germination
14 June	5	0	20 May	5	0
24 June	15	1—Leaf development	4 June	20	1—Leaf development
29 June	20	2—Tillering	14 June	30	2—Tillering
4 July	25	2	24 June	40	2
14 July	35	2	4 July	50	2
19 July	40	2	9 July	55	2
24 July	45	2	14 July	60	3—Stem elongation
29 July	50	3—Stem elongation	29 July	75	4—Booting
3 August	55	3	8 August	85	5—Inflorescence emergence
8 August	60	3	13 August	90	6—Flowering
18 August	70	4—Booting	23 August	100	6
23 August	75	5—Inflorescence emergence	28 August	105	7—Development of grain
2 September	85	6—Flowering	22 September	130	9—Senescence
7 September	90	6			
27 September	110	7—Development of grain			
2 October	115	8—Ripening			
12 October	125	9—Senescence			

**Table 6:** Spectral characteristics and spatial resolutions of Sentinel-2 bands used in study [18].

Spectral Band Name	Wavelength (nm)	Spatial Resolution (m)
B02—Blue	458–523	10
B03—Green	543–578	10
B04—Red	650–680	10
B05—Vegetation Red Edge 1	698–713	20
B06—Vegetation Red Edge 2	733–748	20
B07—Vegetation Red Edge 3	773–793	20
B08—NIR	785–899	10
B8A—NIR narrow	855–875	20
B11—SWIR 1	1565–1655	20
B12—SWIR 2	2100–2280	20

**Table 7:** Spectral characteristics and spatial resolutions of Sentinel-2 bands used.

Vegetation Index	Equation	Equation	Reference
Normalized Difference Vegetation Index (NDVI)	$\frac{\left(B08-B04\right)}{\left(B08+B04\right)}$	(1)	[19]
Green NDVI (GNDVI)	$\frac{\left(B08-B03\right)}{\left(B08+B03\right)}$	(2)	[20]
Normalized Difference Red Edge (NDRE)	$\frac{\left(B08-B05\right)}{\left(B08+B05\right)}$	(3)	[21]
Normalized Difference Water Index (NDWI)	$\frac{\left(B08-B11\right)}{\left(B08+B11\right)}$	(4)	[22]

**Table 8:** Average water depth and yield for each plot in 2022 and 2023. Different letters in the same column indicate statistically significant differences in the LSD test (*p* < 0.05) between plots.

2022			2023		
Field	Height (cm)	Yield (kg·ha^−1^)	Field	Height (cm)	Yield (kg·ha^−1^)
1	11.41 a	7333.5 a	1	4.34 e	5988.23 f
2	5.84 d	6574.04 d	2	5.98 c	9003.42 a
3	8.24 b	7244.56 ab	3	5.45 d	7898.82 c
4	5.78 d	5425.50 e	4	12.40 a	6950.02 d
5	7.20 c	7208.03 b	5	12.22 a	6623.03 e
6	8.07 b	7124.09 b	6	9.91 b	8316.22 b
7	8.12 b	6772.18 c			
*p* Value	<0.01	<0.01	*p* Value	<0.01	<0.01
Average	8.31	6961.24	Average	6.94	7325.23
Standard deviation	2.14	571.29	Standard deviation	3.12	1254.32
Variation coefficient (%)	25.77	8.21	Variation coefficient (%)	44.94	17.12

## Data Availability

The original contributions presented in this study are included in the article and Supplementary Materials. Further inquiries can be directed at the corresponding author.

## Supplementary Materials

The following supporting information can be downloaded at https://www.mdpi.com/article/10.3390/s25154860/s1, Table S1: Analysis of the linear correlation between water depth and vegetation indices. The correlation coefficient (r) and the coefficient of determination (R^2^) are used to evaluate the linear regression in year 2022 (a) and 2023 (b).
