# Supplementary material for: Assessment of Water Depth Variability and Rice Farming Using Remote Sensing
Source: Sensors (Basel). 2025 Aug 7;25(15):4860. doi: 10.3390/s25154860 (PMC12349411; doi:10.3390/s25154860)
Supplement: Supplementary file 1 [file sensors-25-04860-s001.zip › sensors-3709058-supplementary.pdf]

This Table is added as Supplementary Material to clarify the study of linear correlation between the water depth and the vegetation indices used in the paper.

Table S1. Analysis of the linear correlation between water depth and vegetation indices. *The correlation coefficient (r) and the coefficient of determination (R^2) are used to evaluate the linear regression in year 2022 (a) and 2023 (b).*

a)

**Year 2022**	**NDVI**		**GNDVI**		**NDRE**		**NDWI**	
**DAS**	r	R^2	r	R^2	r	R^2	r	R^2
0	-0,55	0,30	-0,46	0,21	0,07	0,00	-0,05	0,00
5	-0,16	0,02	-0,17	0,03	0,02	0,00	0,02	0,00
15	-0,11	0,01	-0,41	0,16	0,13	0,02	0,06	0,00
20	-0,46	0,21	-0,51	0,26	0,09	0,01	0,59	0,34
25	-0,43	0,18	-0,51	0,26	-0,10	0,01	-0,46	0,22
35	-0,59	0,34	-0,61	0,37	-0,05	0,00	0,59	0,34
40	-0,35	0,12	-0,34	0,11	-0,14	0,02	-0,45	0,20
45	-0,21	0,05	-0,24	0,06	-0,08	0,01	-0,52	0,27
50	-0,05	0,00	-0,06	0,00	-0,09	0,01	-0,27	0,07
55	0,04	0,00	0,11	0,01	-0,10	0,01	-0,08	0,01
60	0,13	0,02	0,32	0,10	-0,09	0,01	-0,07	0,01
70	0,23	0,05	0,27	0,08	-0,02	0,00	-0,13	0,02
80	0,16	0,03	0,22	0,05	-0,04	0,00	-0,18	0,03
85	0,08	0,01	0,06	0,00	-0,08	0,01	0,16	0,03
90	0,00	0,00	-0,04	0,00	-0,09	0,01	0,00	0,00
110	-0,30	0,09	-0,13	0,02	-0,07	0,01	0,13	0,02
115	-0,16	0,03	-0,06	0,00	-0,08	0,01	-0,09	0,01

b)

Year 2023	NDVI		GNDVI		NDRE		NDWI	
**DAS**	r	R^2	r	R^2	r	R^2	r	R^2
0	0,21	0,05	0,50	0,25	-0,51	0,26	-0,51	0,26
5	-0,48	0,23	-0,41	0,17	-0,02	0,00	-0,35	0,12
20	-0,31	0,09	-0,16	0,03	0,02	0,00	0,16	0,03
25	-0,59	0,34	-0,55	0,30	0,16	0,02	-0,50	0,25
30	-0,17	0,03	-0,27	0,07	0,27	0,07	0,41	0,17
40	-0,38	0,14	-0,39	0,16	0,33	0,11	-0,12	0,01
50	-0,54	0,29	-0,54	0,29	0,42	0,17	-0,19	0,04
55	-0,43	0,18	-0,50	0,25	0,53	0,28	-0,47	0,22
60	-0,15	0,02	-0,28	0,08	0,29	0,08	-0,64	0,41
65	-0,13	0,02	-0,28	0,08	0,35	0,13	-0,49	0,24
75	-0,20	0,04	-0,32	0,10	0,38	0,15	-0,40	0,16
80	-0,53	0,26	-0,54	0,29	0,21	0,05	-0,60	0,36
85	0,25	0,06	0,05	0,00	0,49	0,24	-0,43	0,19
90	0,53	0,28	0,52	0,27	0,38	0,15	-0,21	0,04
95	-0,49	0,24	-0,49	0,24	0,38	0,15	-0,62	0,38
100	0,28	0,08	0,03	0,00	0,35	0,12	-0,36	0,13
105	0,23	0,05	-0,08	0,01	0,42	0,18	-0,36	0,13
115	-0,48	0,23	-0,51	0,26	0,26	0,07	-0,67	0,45
125	0,31	0,10	0,33	0,11	0,19	0,04	0,02	0,00
130	-0,05	0,00	-0,17	0,03	0,55	0,31	0,00	0,00
135	0,44	0,19	0,39	0,15	0,15	0,02	0,55	0,31
140	0,52	0,27	0,35	0,12	0,18	0,03	0,37	0,14
